# E-Selectin Gene Polymorphisms and Essential Hypertension in Asian Population: An Updated Meta-Analysis

**DOI:** 10.1371/journal.pone.0102058

**Published:** 2014-07-08

**Authors:** Gaojun Cai, Bifeng Zhang, Weijin Weng, Ganwei Shi, Sheliang Xue, Yanbin Song, Chunyan Ma

**Affiliations:** 1 Department of Cardiology, Wujin hospital affiliated to Jiangsu University, Changzhou, Jiangsu Province, China; 2 Department of Pathology and Molecular Medicine, McMaster University, Ontario, Canada; Children’s National Medical Center, Washington, United States of America

## Abstract

**Objective:**

Epidemiological studies have shown that E-selectin gene polymorphisms (A561C and C1839T) may be associated with essential hypertension (EH), but the results are conflicting in different ethnic populations. Thus, we performed this meta-analysis to investigate a more authentic association between E-selectin gene polymorphisms and the risk of EH.

**Methods:**

We searched the relevant studies for the present meta-analysis from the following electronic databases: PubMed, Embase, Cochrane Library, Google Scholar, Web of Science, Wanfang Data, and China National Knowledge Infrastructure (CNKI). Odds ratios (OR) with 95% confidence interval (CI) were used to evaluate the strength of the association between E-selectin gene polymorphisms and EH susceptibility. The pooled ORs were performed for dominant model, allelic model and recessive model. The publication bias was examined by Begg’s funnel plots and Egger’s test.

**Results:**

A total of eleven studies met the inclusion criteria. All studies came from Asians. Ten studies (12 cohorts) evaluated the A561C polymorphism and EH risk, including 2,813 cases and 2,817 controls. The pooled OR was 2.280 (95%CI: 1.893–2.748, *P*<0.001) in dominant model, 5.284 (95%CI: 2.679–10.420, *P*<0.001) in recessive model and 2.359 (95%CI: 1.981–2.808, *P* = 0.001) in allelic model. Four studies (six cohorts) evaluated C1839T polymorphism and EH risk, including 1,700 cases and 1,681 controls. The pooled OR was 0.785 (95%CI: 0.627–0.983, *P* = 0.035) in dominant model, 1.250 (95%CI: 0.336–4.652, *P* = 0.739) in recessive model and 0.805 (95%CI: 0.649–0.999, *P* = 0.049) in allelic model.

**Conclusion:**

The current meta-analysis concludes that the C allele of E-selectin A561C gene polymorphism might increase the EH risk in Asian population, whereas the T allele of E-selectin C1839T gene polymorphism might decrease the EH risk.

## Introduction

Essential hypertension (EH) is a complex multifactorial disease caused by genetic and environmental factors [1], [2] Epidemiological data shows that there are about one billion EH patients in the world. At present, EH is becoming the major risk factor increasing the mortality and the morbidity of coronary heart disease (CHD), ischemic stroke (IS), chronic heart failure and chronic renal failure. Similar as CHD and some type of IS, EH is characterized by chronic inflammation. The inflammation plays an important role in the pathogenesis and the maintenance of EH, which has received increased attention in the past few years. At the same time, a growing body of evidence indicates that oxidative stress is involved in the pathophysiological mechanism and development of hypertension [3]. The levels of oxidative stress markers are significantly higher in hypertensive patients.

E-selectin, a cell-surface single chain glycoprotein, was firstly identified in 1985. It is a member of adhesion molecule, also known as endothelial leukocyte adhesion molecule-1(ELAM-1), CD62 antigen-like family member E (CD62E) and SELE. E-selectin is expressed only by activated endothelial cells, and is different from other selectins. The concentration of E-selectin is very low in resting endothelial cells. When vascular endothelial cells are stimulated by inflammatory factors, the expression of E-selectin is greatly increased. Previous researches have revealed that the level of E-selectin is significantly higher in EH group than in controls [4] and is positively associated with diastolic blood pressure values in children with Type 1 diabetes mellitus (T1DM) [5]. High blood pressure may activate and damage the endothelial cells, which results in the increase of E-selectin. Subsequently, the adhesion and aggregation between leukocytes and endothelial cells increased which can change the structure and function of microcirculation. These changes in microcirculation may promote the increase of blood pressure. In addition, E-selectin also has a pro-angiogenesis effect itself.

The gene encoding E-selectin is located in human chromosome 1q22-q25 and contains 14 exons spanning about 13 kilobases of the human genome. Several E-selectin gene polymorphisms have been identified recently. In 1994, Wenzel *et al.* [6] firstly identified the E-selectin A561C gene polymorphism, which exists in exon 4 of the gene. This variation in the EGF domain may be relevant to ligand binding. The polymorphism of C1839T also exists in the exon of the gene, which may play an important role in the correct membrane anchoring of the protein. Studies have shown that E-selectin A561C and C1839T polymorphisms might be associated with inflammation related diseases, such as severe atherosclerosis, CHD and some type of IS [7], [8]. To date, a number of epidemiological studies were performed to explore the relationships between E-selectin A561C and/or C1839T polymorphisms and risk of EH, but the results were conflicting in different ethnic populations [9]–[19]. Several studies concluded that these polymorphisms might increase the susceptibility to EH [10]–[13], [15], [20]. But other studies found the controversial conclusion [9], [19]. In addition, the relatively small sample size in each study is relatively small. Thus, we performed this meta-analysis, including eleven studies and aimed to derive a more precise association of the E-selectin gene polymorphisms (A561C and C1839T) and risk of EH.

## Methods

### Studies selection

We searched the relevant studies for the present meta-analysis (last search was updated on January 1, 2014) from the following electronic databases: PubMed, Embase, Cochrane Library, Google Scholar, Web of Science, Wanfang Data (http://www.wanfangdata.com.cn), and China National Knowledge Infrastructure (CNKI). The following search terms were used in the electronic searches: “E-selectin *or* endothelial leukocyte adhesion molecule-1 *or* ELAM-1, *or* CD62 antigen-like family member E *or* CD62E” and “variant *or* mutation *or* gene *or* polymorphism” and “essential hypertension”. We also checked the references of relevant studies to minimize the omissions.

Studies included in this meta-analysis must meet all the following criteria: (a) case-control study evaluating the relationship between E-selectin polymorphism and EH; (b) having the clear original data of genotypic and/or allelic frequencies; (c) papers must be written in either Chinese or English; (d) no restriction on the sample size; (e) hypertension was defined as systolic blood pressure ≥140 mmHg and/or diastolic blood pressure ≥90 mmHg or treatment with antihypertensive medication, and only essential hypertension was included (secondary hypertension excluded). If we were able to obtain several similar data from one research center, we retained the most comprehensive study.

### Data extraction

A standard protocol was used to record the original data. All relevant studies were read carefully by two investigators (Cai and Zhang) and the original data were extracted independently. If there was any doubtful point, the disagreement was resolved by discussion between the two authors. The characteristics of each study were extracted, including the name of the first author, year of publication, average age, gender, region, ethnicity, numbers of cases and controls, numbers of genotypes in cases and controls, diagnostic criteria of EH and the genotyping methods. If the study did not offer the original data which we need, we tried our best to contact the correspondent author by telephone or E-mail.

**Figure 3:**
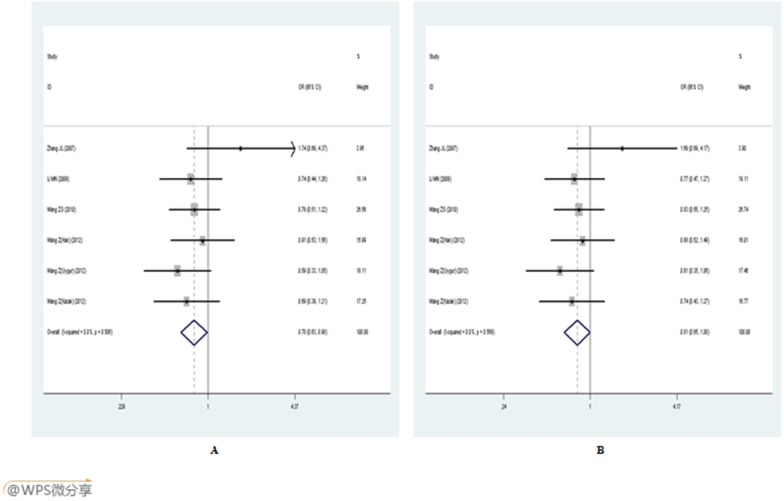
Forest plot of E-selection gene C1839T polymorphism and EH risk. A: dominant genetic model (CT+TT *vs.* CC); B: allelic genetic model (T *vs.* C).

For C1839T polymorphism, the T allele was found to have a significantly protective effect against EH risk. The pooled OR was 0.785 (95%CI: 0.627–0.983, *P* = 0.035) in dominant model and 0.805 (95%CI: 0.649–0.999, *P* = 0.049) in allelic model (**Fig. 3**). When the studies were stratified by ethnicity, genotyping methods and total sample size, the positive results only existed in the Taqman-PCR subgroup. The pooled OR was 0.747 (95%CI: 0.577–0.967, *P* = 0.027) in dominant model and 0.769 (95%CI: 0.599–0.986, *P* = 0.038) in allelic model.

### Data analysis

Hardy-Weinberg equilibrium (HWE) for the E-selectin genotype distributions of control groups was determined by the Fisher’s exact test. Pooled odds ratios (OR) with 95% confidence interval (CI) were used to evaluate the strength of association between the E-selectin gene polymorphisms and the EH susceptibility. We calculated the pooled ORs by dominant model (for A561C: CC+CA versus AA; for C1839T: TT+CT versus CC), allelic model (for A561C: C versus A; for C1839T: C versus T) and recessive model (for A561C: CC versus CA+AA; for C1839T: TT versus CT+CC). The subgroup analyses were carried out by ethnicity (Han or others), total sample size (less than 600 or more than 600) and genotyping methods (PCR-RFLP or Taqman PCR). The Q-test and *I* ^2^ statistics were used to assess the heterogeneity among studies. The fixed effect model (the Mantel-Haenszel method) was adopted to calculate the pooled results if the heterogeneity was not significant (I^2^<50%, *P*>0.10). Otherwise, the random-effect model (the Dersimonian-Laird method) was applied [21]. The analysis of influence was checked by one-way sensitivity analysis and also by calculating the results again when omitting study deviating from HWE. The potential publication bias between studies was established by Begg’s funnel plots. Egger’s test on the natural logarithm scale of the OR was used to examine the funnel plots asymmetry.

All statistical analyses were performed by using STATA software version 12.0 for Windows (StataCorp LP, College Station, Texas 77845 USA). A *P* value <0.05 (two-sided) was considered statistically significant.

## Results

### Characteristics of studies

**Figure 1:**
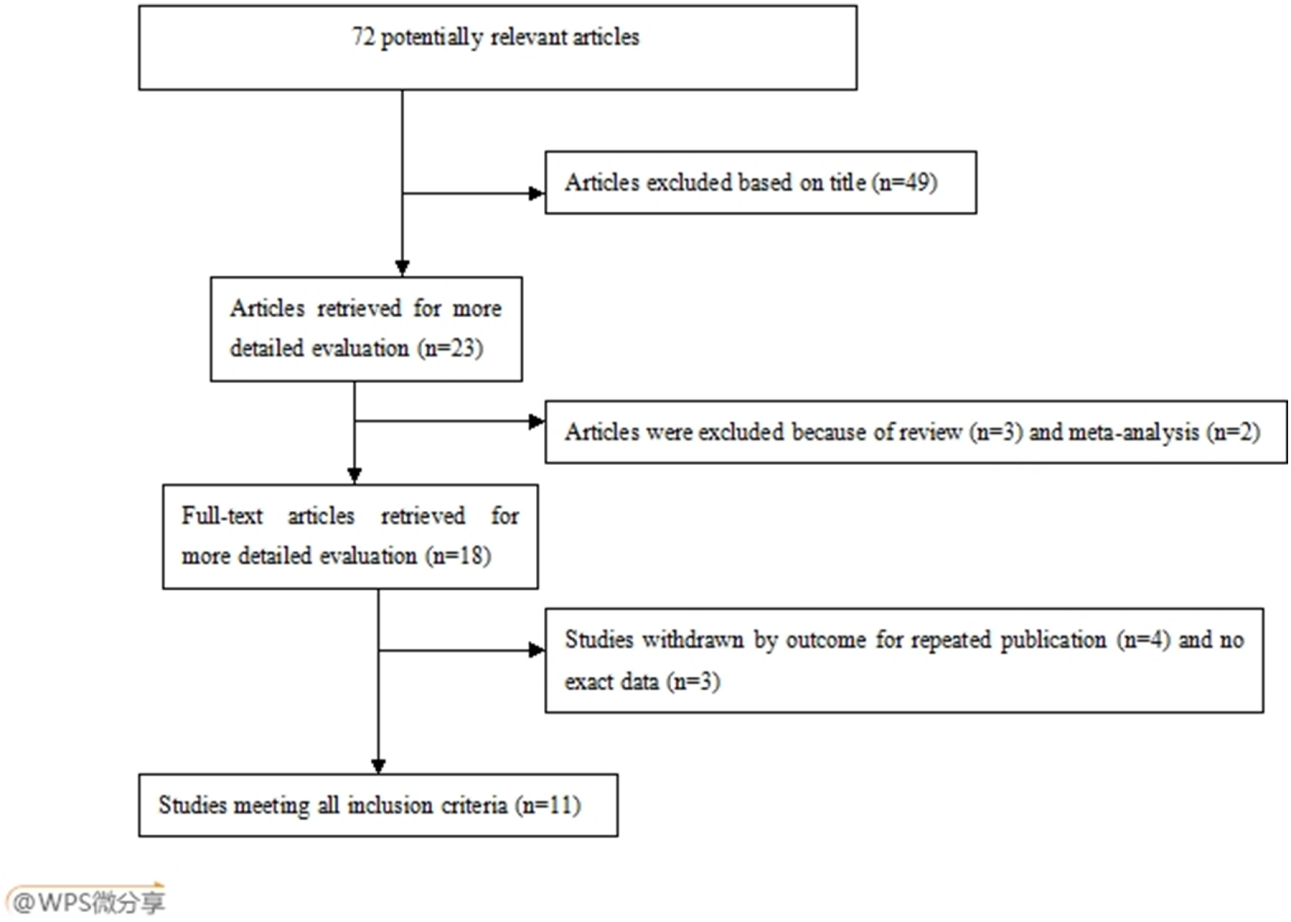
Flow diagram of articles selection process for E-selectin gene polymorphisms and EH risk meta-analysis.

Through the literature search, 72 eligible papers were identified, of which 61 papers were excluded. Of the 61 excluded studies, three papers were reviews, two papers were meta-analysis, four studies repeated prior researches, three studies lacked of reported data and failed to obtain relevant information, 49 studies were unrelated to the E-selectin A561C or C1839T gene polymorphisms and EH. The flow diagram of articles selection process is listed in **Fig. 1**. A total of eleven studies met the inclusion criteria. Because one article [16] contained three ethnic groups (Han, Uygur and Kazak) with three independent results, we analyzed it as three separated cohorts for each polymorphism. Ten studies (twelve cohorts) evaluated A561C polymorphism and EH risk, including 2,813 cases and 2,817 controls. The C allelic frequency ranged from 1.12% to 8.28%. Four studies (six cohorts) evaluated C1839T polymorphism and EH risk, including 1,700 cases and 1,681 controls. The T allelic frequency ranged from 3.36% to 13.16%. All studies came from Asians. One study was conducted in Asian Indian populations and others were conducted in Chinese populations. The ethnicities included Han, Hani, Uygur, Kazak and Indian. Two genotyping methods were used, including polymerase chain reaction-restriction fragment length polymorphism (PCR-RFCA) and Taqman PCR. Age was matched in all of studies. The diagnostic criteria of EH was appropriated in all of these studies. The genotype distribution in one study deviated from HWE [11]. The main characteristics of studies are shown in **Table 1**.

**Table 1:** Characteristics of the investigated studies of the association between E-selectin gene polymorphisms (A561C, C1839T) and EH.

SNP	First author	Year	Region	Ethnicity	Age	Sample size		Genotype(case group)			Genotype(control group)			GenotypingMethods	HWE(*P*)
						Case	Control	MM	Mm	mm	MM	Mm	mm		
**rs5361** **(A561C)**	Li MN [9]	2009	Yunnan (China)	Hani	52.2±10.5/50.6±9.7	172	133	149	22	1	120	13	0	PCR-RFCA	0.553
	Zheng WW [10]	2009	Xinjiang (China)	Kazak	47.4±11.4/44.1±11.1	150	150	117	33	0	132	18	0	PCR-RFCA	0.434
	Chen HL [11]	2005	Beijing (China)	Han	46.1±12.5/42.2±14.7	347	315	323	15	9	305	6	4	PCR-RFCA	0.000
	Song Y [12]	2007	Guangdong (China)	Han	-	105	94	94	10	1	91	3	0	PCR-RFCA	0.875
	Liu ZC [13]	2006	Heibei (China)	Han	61.3±8.7/59±5.1	95	101	75	18	2	92	8	1	PCR-RFCA	0.112
	Li Y [14]	2003	Hubei (China)	Han	68.5±6.3/67.2±5.5	176	182	158	18	0	167	15	0	PCR-RFCA	0.562
	Srivastava [15]	2012	Delhi (Indian)	Asian Indian	51.6±7.2/49.7±10.4	240	290	142	94	4	244	44	2	PCR-RFCA	0.991
	Wang Z [16]	2012	Xinjiang (China)	Han	49.1±10.7/48.1±9.9	366	349	329	24	13	339	10	0	TaqMan PCR	0.786
	Wang Z [16]	2012	Xinjiang (China)	Uygur	54.4±8.0/55.9±8.7	309	299	273	22	14	276	23	0	TaqMan PCR	0.489
	Wang Z [16]	2012	Xinjiang (China)	Kazak	50.0±13.3/50.6±14.5	264	275	237	26	1	255	20	0	TaqMan PCR	0.055
	Wang ZG [17]	2010	Beijing (China)	Han	53.8±8.0/51.5±8.9	490	495	447	38	5	474	21	0	TaqMan PCR	0.630
	Zhang JL [18]	2007	Yunnan (China)	Yi	47.1±10.4/45.7±7.5	99	134	91	8	0	131	3	0	PCR-RFCA	0.896
**rs5355** **(C1839T)**	Li MN [19]	2009	Yunnan (China)	Hani	52.2±10.5/50.6±9.7	172	133	136	36	0	98	35	0	PCR-RFCA	0.081
	Wang Z [16]	2012	Xinjiang (China)	Han	49.1±10.7/48.1±9.9	368	349	340	28	0	320	28	1	TaqMan PCR	0.644
	Wang Z [16]	2012	Xinjiang (China)	Uygur	54.4±8.0/55.9±8.7	307	299	286	21	0	266	33	0	TaqMan PCR	0.313
	Wang Z [16]	2012	Xinjiang (China)	Kazak	50.0±13.3/50.6±14.5	264	272	241	22	1	239	33	0	TaqMan PCR	0.287
	Wang ZG [17]	2010	Beijing (China)	Han	53.8±8.0/51.5±8.9	490	495	450	37	3	445	48	2	TaqMan PCR	0.547
	Zhang JL [18]	2007	Yunnan (China)	Yi	47.1±10.4/45.7±7.5	99	134	88	11	0	125	9	0	PCR-RFCA	0.688

Abbreviations: M, major allele; m, minor allele; SNP, single nucleotide polymorphism; PCR-RFLP, polymerase chain reaction–restriction fragment length polymorphism; HWE, Hardy-Weinberg equilibrium.

### Quantitative synthesis

**Figure 2:**
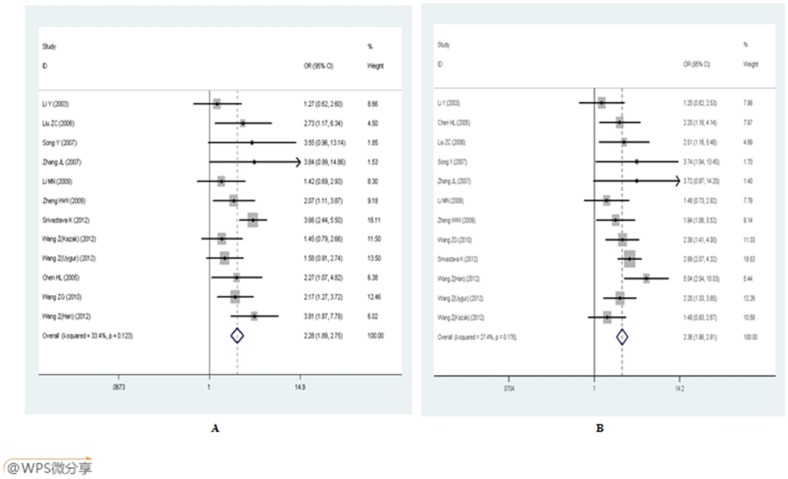
Forest plot of E-selection gene A561C polymorphism and EH risk. A: dominant genetic model (AC+CC *vs.* AA); B: allelic genetic model (C *vs.* A).

The main results of the meta-analyses are listed in **Table 2**. Because each individual polymorphism lacked of heterogeneity, we adopted the fixed effect model to calculate the pooled ORs. Overall, the E-selectin gene polymorphisms were significantly associated with EH susceptibility. For A561C polymorphism, the pooled OR was 2.280 (95%CI: 1.893–2.748, *P*<0.001) in dominant model, 5.284 (95%CI: 2.679–10.420, *P*<0.001) in recessive model and 2.359 (95%CI: 1.981–2.808, *P* = 0.001) in allelic model. The E-selectin A561C polymorphism significantly increased the EH susceptibility (**Fig. 2**). When the studies were stratified by ethnicity, genotyping methods and total sample size, the positive results existed in all of these stratified subgroups except less than 600 subgroup and PCR-RFLP subgroup in recessive group.

**Table 2:** Summary of meta-analysis of association of E-selectin gene polymorphisms and EH risk.

SNP	Total orsubgroupanalysis	Studies(Cases/Controls)	Allelic model			Dominant model			Recessive model		
			OR (95%CI)	*P*_OR_	*P* forheterogeneity	OR (95%CI)	*P*_OR_	*P* forheterogeneity	OR (95%CI)	*P*_OR_	*P* forheterogeneity
**A561C**	**Total**	12 (2813/2817)	2.359 (1.981–2.808)	**0.001**	0.176	2.280 (1.893–2.748)	**<0.001**	0.123	5.284 (2.679–10.420)	**<0.001**	0.520
	**Ethnicity**										
	Han	6 (1579/1536)	2.557 (1.939–3.373)	**<0.001**	0.138	2.365 (1.758–3.181)	**<0.001**	0.393	4.684 (2.008–10.932)	**<0.001**	0.390
	Others	6 (1234/1281)	2.230 (1.781–2.792)	**<0.001**	0.260	2.224 (1.750–2.828)	**<0.001**	**0.046**	6.468 (2.067–20.244)	**0.001**	0.401
	**Total** **sample size**										
	Less than 600	8 (1301/1359)	2.175 (1.743–2.714)	**<0.001**	0.204	2.307 (1.824–2.919)	**<0.001**	0.077	2.480 (0.815–7.546)	0.110	1.000
	More than 600	4 (1512/1458)	2.672 (2.011–3.549)	**<0.001**	0.221	2.237 (1.648–3.037)	**<0.001**	0.299	7.450 (3.067–18.095)	**<0.001**	0.101
	**Genotyping** **methods**										
	PCR-RFLP	8 (1384/1399)	2.299 (1.837–2.877)	**<0.001**	0.340	2.477 (1.743–3.156)	**<0.001**	0.175	2.227 (0.962–5.158)	0.062	1.000
	Taqman-PCR	4 (1429/1418)	2.450 (1.857–3.233)	**<0.001**	0.065	2.025 (1.513–2.711)	**<0.001**	0.171	17.609 (4.225–73.385)	**<0.001**	0.702
**C1839T**	**Total**	6 (1700/1682)	0.805 (0.649–0.999)	**0.049**	0.569	0.785 (0.627–0.983)	**0.035**	0.506	1.250 (0.336–4.652)	0.739	0.587
	**Ethnicity**										
	Han	2 (858/844)	0.848 (0.612–1.173)	0.319	0.856	0.835 (0.595–1.173)	0.299	0.696	0.992 (0.226–4.364)	0.992	0.400
	Others	4 (842/838)	0.774 (0.580–1.032)	0.081	0.300	0.748 (0.553–1.010)	0.058	0.270	3.102 (0.126–76.499)	0.489	-
	**Total** **sample size**										
	Less than 600	3 (535/539)	0.847 (0.605–1.187)	0.336	0.265	0.819 (0.575–1.168)	0.270	0.219	3.102 (0.126–76.499)	0.489	-
	More than 600	3 (1165/1143)	0.778 (0.587–1.029)	0.079	0.588	0.763 (0.570–1.020)	0.068	0.553	0.992 (0.226–4.364)	0.992	0.400
	**Genotyping** **methods**										
	PCR-RFLP	2 (271/267)	0.928 (0.602–1.430)	0.734	0.134	0.919 (0.581–1.454)	0.719	0.117	-	-	-
	Taqman-PCR	4 (1429/1415)	0.769 (0.599–0.986)	**0.038**	0.778	0.747 (0.577–0.967)	**0.027**	0.734	1.250 (0.336–4.652)	0.739	0.587

### An analysis of influence

**Figure 4:**
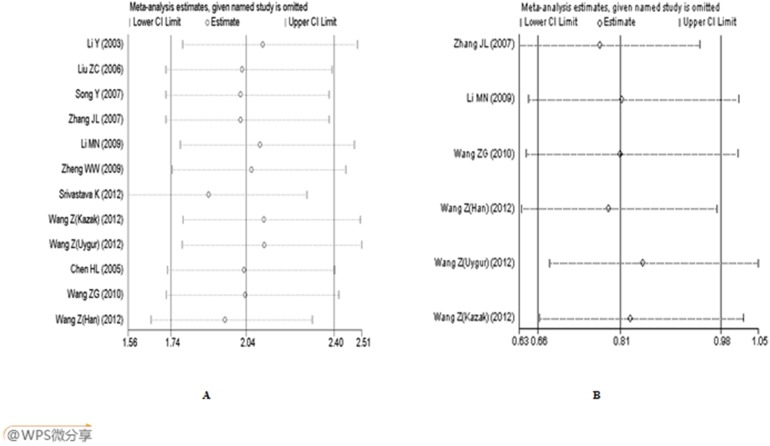
Analysis of influence of individual study on the pooled estimate in dominant model in overall population. A: A561C (AC+CC *vs.* AA); B: C1839T (CT+TT *vs.* CC).

We performed the sensitivity analysis by calculating pooled ORs again when omitting one study at a time. For each polymorphism, the results didn’t show any significant difference when any study was omitted. This indicated that each single study didn’t influence the stability of the entire study. **Fig. 4**. shows the sensitivity analysis in dominant model in overall population. For A561C polymorphism, the sensitivity analysis was also performed by deleting the study deviated from the HWE, and the pooled OR did not change significantly, which also indicated that our results were statistically reliable.

### Publication bias evaluation

**Figure 5:**
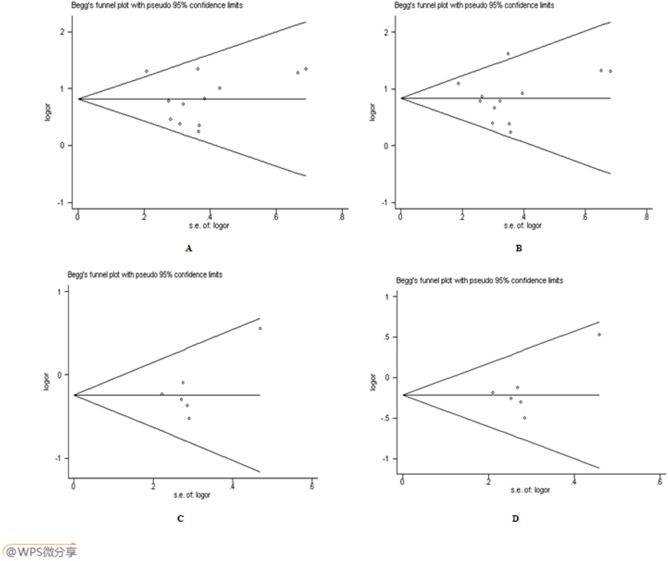
Funnel plot for studies of the association between E-selection gene polymorphisms and EH. A: A561C (AC+CC *vs.* AA); B: A561C (C *vs.* A); C: C1839T (CT+TT *vs.* CC); D: C1839T (T *vs.* C).

Both Begg’s funnel plot and Egger’s test were performed to evaluate the publication bias of the literatures. **Fig. 5**. displays the funnel plots which examined the A561C and C1839T polymorphisms and the overall EH risk included in this meta-analysis. For A561C, the effect size was asymmetrically distributed with publication bias visually present, which was confirmed by Egger’s test (*P* = 0.034 for allelic model). Whereas, no significant publication bias was found in the dominant model (*P* = 0.054) and in recessive model (*P* = 0.974). For C1839T, there was no significant publication bias according to the visual assessment of funnel plot and Egger’s test (*P* = 0.108 for dominant model, 0.126 for allelic model, 0.768 for recessive model).

## Discussion

To our knowledge, the current meta-analysis was the first study to investigate the relationship between E-selectin gene polymorphisms and EH risk. Our study suggests that E-selectin gene A561C and C1839T polymorphisms might be associated with EH risk.

CHD and some type of IS are both characterized by chronic inflammation. EH also has a close association with inflammation. Studies show that several genetic polymorphisms are related to EH and genetic factors maybe account for 30% to 50% of the causes of the variation in blood pressure [1].

In 2011, Sun *et al.* [22] performed a meta-analysis to explore the relationship between E-selectin polymorphism and IS. They found that E-selectin A561C polymorphism was significantly associated with IS susceptibility and that both the AC+CC genotype and the C allele may be associated with IS susceptibility. In 2013, Wang *et al.* [23] also carried out a meta-analysis to investigate the relationship between E-selectin A561C polymorphism and the disease. A total of 22 papers were included in their study and they found E-selectin A561C polymorphism was significantly associated with CHD.

In 2012, Srivastava *et al*. [15] evaluated the relationship between E-selectin gene A561C polymorphism and the EH risk in Asian Indians. They found that individuals with C allele increased the risk to develop EH by 2.8 times at 95% CI after age and sex were adjusted. Chen *et al*. [11] explored the relationships between E-selectin A561C polymorphism and EH and ambulatory blood pressure in Chinese population. They concluded that patients with AC-CC genotype and C allele had higher diastolic blood pressure and mean arterial pressure. The risk of EH for people carrying allele C was 2.197 times of that of allele A carriers. At the same time, they also found that AC-CC genotype carriers compared with the AA genotype carriers have a greater body mass index (BMI), higher fasting blood glucose (FBG). Results suggested that E-selectin A561C polymorphism may be associated with insulin resistance and hyperinsulinemia. Song *et al*. [12] also found the risk of hypertension in AC-CC genotype carriers was 3.55 times of the risk in carriers of AA genotype and hypertensive patients with AA-AC genotype were prone to left ventricular hypertrophy.

Compared with A561C polymorphism, the number of study about the connection between E-selectin C1839T polymorphism and EH was relatively small. In 2004, Marteaua *et al*. [20] studied the relationship between C1839T polymorphism and longitudinal blood pressure changes in a Stanislas cohort. They concluded that no association was found between C1839T polymorphism and blood pressure at the beginning of study. But 5 years later, there was a great increase in both systolic and diastolic blood pressure in the subjects who carry the T allele and have a BMI greater than 25 kg/m^2^.

By contrast, Li *et al*. [9] found there was no statistical significance between E-selectin A561C polymorphism and EH in Chinese Hani population. At the same time, they found there was a negative relationship between E-selectin C1839T polymorphism and EH, but the result was not statistical significant [19]. In Xinjiang area, Wang *et al*. [16] investigated the relationship between E-selectin gene polymorphisms and EH in Han, Kazakh and Uygur populations. They also found there was no significant association between C1839T polymorphism and EH risk in these populations.

A total of 11 studies included in our meta-analysis. Ten studies (twelve cohorts) evaluated A561C polymorphism polymorphism and EH risk. The C allele of E-selectin A561C gene might increase the EH risk in Asian population (ORs = 2.280, 95%CI: 1.893–2.748, *P*<0.001, in dominant model). For C1839T polymorphism, the present meta-analysis suggested that the T allele of E-selectin C1839T gene polymorphism might decrease the EH risk (ORs = 0.785, 95%CI: 0.627–0.983, *P* = 0.035, in dominant model).

Some limitations still exist in the current research. Firstly, there was inadequate large-scale research on the relationship between EH and E-selectin gene polymorphisms (A561C and C1839T). The total sample size of each study was less 1000. Especially, the number of studies on C1839T polymorphism and EH was only 4 (6 cohorts). Secondly, E-selectin was affected not only by the E-selectin gene polymorphisms but also by environmental factors, for example inflammation. Thirdly, all the studies were conducted in Asian populations (most of them were Chinese population). Studies from other continents should be performed in the future. Most importantly, publication bias existed in the current meta-analysis (for EL A561C gene polymorphism). For example, studies may not have been published, if they failed to demonstrate an association between E-selectin A561C gene polymorphism and EH risk. The publication bias could cause the false positive results. In addition, our study only focused on papers published in the English or Chinese, and therefore some eligible studies written in other languages were not included.

Despite the limitations, the current meta-analysis concluded that the C allele of E-selectin A561C gene polymorphism might increase the EH risk in Asian populations, whereas T allele of E-selectin C1839T gene polymorphism might decrease the EH risk. Given the above limitations, further studies should be performed to clarify the association between the E-selectin gene polymorphisms and EH through multicenter, large-scale, and multi-ethnic studies in the future.

## Supporting Information

Checklist S1 PRISMA Checklist.
(DOC)
